# Immunomodulatory Role of CB2 Receptors in Emotional and Cognitive Disorders

**DOI:** 10.3389/fpsyt.2022.866052

**Published:** 2022-04-15

**Authors:** Alvaro Morcuende, María Salud García-Gutiérrez, Simone Tambaro, Elena Nieto, Jorge Manzanares, Teresa Femenia

**Affiliations:** ^1^Instituto de Neurociencias, Universidad Miguel Hernández-Consejo Superior de Investigaciones Científicas (CSIC), Alicante, Spain; ^2^Redes de Investigación Cooperativa Orientada a Resultados en Salud, Red de Investigación en Atención Primaria de Adicciones, Instituto de Salud Carlos III, Ministerio de Ciencia e Innovación (MICINN) and Fondo Europeo de Desarrollo Regional (FEDER), Madrid, Spain; ^3^Instituto de Investigación Sanitaria y Biomédica de Alicante, Alicante, Spain; ^4^Division of Neurogeriatrics, Center for Alzheimer Research, Department of Neurobiology, Care Sciences and Society, Karolinska Institutet, Stockholm, Sweden

**Keywords:** cannabinoid receptor 2, psychiatry, anxiety, depression, Alzheimer’s disease, inflammation, immunomodulation

## Abstract

Emotional behavior, memory, and learning have been associated with alterations in the immune system in neuropsychiatric and neurodegenerative diseases. In recent years, several studies pointed out the involvement of the cannabinoid receptor 2 (CB2r) in the immune system and the regulation of inflammation. This receptor is widely distributed in different tissues and organs with higher expression in spleen and immune system cells. However, CB2r has also been detected in several brain areas and different brain cell types, such as neurons and glia. These findings suggest that CB2r may closely relate the immune system and the brain circuits regulating inflammation, mood, and cognitive functions. Therefore, we review the studies that may help elucidate the molecular bases of CB2r in regulating inflammation in different brain cells and its role in the pathophysiology of psychiatric and neurodegenerative disorders.

## Introduction

Psychiatric disorders are a heterogeneous group where alterations at the inflammatory level have been identified, relating in some cases to the onset or progression of psychiatric disorders.

Studies reported increased circulating inflammation in individuals with mood disorders characterized by cognitive dysfunction (1–9).

Inflammation is a process that helps repair tissue damage or resolve an infection, in which the rapid response of the innate immune system plays a significant role. Different mediators known as Damage Associated Molecular Patterns (DAMPS) are released into the extracellular space during tissue damage. These DAMPS are recognized by specific receptors, Pattern Recognition Receptors (PRR), composed of several families of receptors. The most important PRRs are the Toll-Like Receptors (TLRs) or NOD-Like Receptors (NLRs). These receptors recognize DAMPS and Pathogen-associated Molecular Patterns (PAMPS) or products associated with oxidative stress such as reactive oxygen species (ROS). Once these PRRs are activated, they initiate an intracellular signaling cascade triggering the release of inflammatory mediators through the Type I interferon and Nuclear Factor Kappa-B (NF-kB) signaling pathways, the most studied pathways of pro-inflammatory cytokine production (10, 11). In an inflammatory process, there is a resolution phase where anti-inflammatory mechanisms such as anti-inflammatory cytokines, maresins, and resolvins are stimulated (12). When this fails, the consequence is chronic and pathological inflammation, but still, the organism will try to compensate for this inflammation to reach homeostasis. In this context, a delicate balance between the levels of pro-inflammatory cytokines (e.g., L-1β, IL-6, and TNF-α) and anti-inflammatory (e.g., IL-4, IL-10, IL-11, and IL-13) is necessary for a proper resolution of the inflammation process. These signals are regulated upon the phenotype acquired of the immune cells and microglia at specific time windows (i.e., M1 pro-inflammatory phenotype or M2 anti-inflammatory phenotype) (13, 14).

Noteworthy, the release of DAMPS and the consequent release of pro-inflammatory cytokines produce a systemic sterile inflammation without the participation of pathogens. This systemic inflammation may promote disruption of the blood-brain barrier, making it more permeable to the passage of DAMPS, pro-inflammatory cytokines, or infiltration of leukocytes and macrophages associated with neuroinflammation (15–17). In this way, sterile inflammation has been linked to various psychiatric illnesses, especially to episodes of major depression or bipolar disorder (8, 10, 11). In this sense, it has been observed in different clinical studies that the administration of non-steroidal anti-inflammatory drugs (NSAIDs), mainly using selective COX-2 inhibitors, can improve the psychiatric pathology, evidencing the close correlation between these diseases and the immune system (18).

In this regard, during the last years, many findings suggested the participation of the endogenous cannabinoid system in the modulation of behavior and inflammation. Therefore, due to the possible importance of inflammation in the onset and development of psychiatric diseases, it is necessary to investigate further the precise mechanisms underlying this process to discover new therapeutic targets.

## The Endogenous Cannabinoid System

The endogenous cannabinoid system (ECS) is composed of several receptors, including the cannabinoid receptor 1 (CB1r) and the cannabinoid receptor 2 (CB2r), endogenous ligands, and enzymes responsible for their synthesis and degradation. In addition, it should be noted that there are other atypical receptors, not CB1/CB2, which include the GPR family receptors. Their effects could be mediated through the activation of different mechanisms, such as gene transcription mediated by peroxisome proliferator-activated receptors (PPARs), transient receptor potential channels (TRP), or vanilloid receptors like TRPV1 (19–21).

The main actions of CB1r and CB2r include inhibition of adenylate cyclase, receptors coupled to G proteins of the Go and Gi type. However, while CB1r and CB2r have a similar affinity for Gi type, CB2r has a lower affinity for Go than Gi (22); inhibition of voltage-dependent calcium channels; activation of MAP kinases; and modulation of potassium channels. Their activation leads to cell physiology changes as diverse as synaptic function, gene transcription, and cell migration (19, 21, 23, 24). However, cannabinoid receptors combine among themselves; Callen et al. (25) report the presence of CB1-CB2 receptor heteromers in different brain areas, showing a bidirectional cross-antagonism phenomenon between them.

Regarding the distribution of endocannabinoid receptors, CB1r is widely distributed in the central nervous system (CNS), in brain regions such as the cortex, basal ganglia, hippocampus, cerebellum, dorsal and ventral striatum, globus pallidus, and substantia nigra, among other areas. These receptors are found in neurons and glia and seem to play an important role in motivation and cognition, involved in GABAergic and glutamatergic pathways (19, 21, 23, 24). On the other hand, CB2r was initially discovered in the spleen, suggesting a role in immunity. It was believed to be the primary expression site for a long time and with no expression in the brain. Since the CB2R expression in the healthy brain is low and the available tools have important limitations (i.e., issues with antibodies specificity). It was classically thought to be expressed in microglial cells and vascular elements and to increase in large numbers in the presence of tissue damage or neuroinflammation (19, 21, 23, 24). However, it has also been found in basal conditions in neurons and brain areas highly involved in emotional, rewarding, and cognitive behaviors, such as the ventral tegmental area (VTA), nucleus accumbens, amygdala, or hippocampus. Therefore, it is suggested that it may have an essential role in several brain disorders related to these behaviors (24).

Among the endogenous cannabinoids (EC), the two most studied ones are the arachidonoylethanolamine or anandamide (AEA) and the 2-arachidonoylglycerol (2-AG) (23). Typically, the endogenous ligands are derived from membrane phospholipids and therefore are not stored in synaptic vesicles, in contrast to other neurotransmitters (19, 23). However, other EC ligands have been characterized, such as lysophosphatidylinositol (LlI), virodhamine, and noladin ether.

In the synthesis of EC, both AEA and 2-AG are derived from arachidonic acid, the primary source of arachidonic acid, the omega-6 polyunsaturated fatty acids (PUFAs). The AEA is produced from the precursor *N*-arachidonoyl-phosphatidyl-ethanol (NAPE). It is thought that AEA is obtained from this precursor and by four possible routes, being NAPE phospholipase D (NAPE-PLD), a direct and well-characterized route (19, 23). Although the synthesis of 2-AG appears to be simple, the majority comes from the sequential hydrolysis of arachidonoyl-phosphatidylinositol-bisphosphate by a phospholipase (PLCβ) followed by hydrolysis of the resulting diacylglycerol by the enzyme diacylglycerol lipase (DAGL) (19, 23). The importance of 2-AG as an intermediate metabolite in lipid synthesis must be emphasized. The primary source of arachidonic acid for the biosynthesis of prostaglandins (19, 23) stands out for their involvement as an inflammatory mediator. Two isoforms of DAGL, α, and β, have been characterized. In animal studies, it was proposed that DAGLα is a crucial enzyme for neuronal plasticity in adults.

The metabolization of AEA in the central nervous system is carried out mainly by the fatty acid aminohydrolase or FAAH, which is found in postsynaptic neurons and can also degrade various fatty acid derivatives. In addition, anandamide can be metabolized by cyclooxygenase-2 (COX-2) or *N*-acylethanolamine amidase (NAAA) (19, 21, 23). The degradation will be carried out mainly by the monoacylglycerol lipase (MAGL) located in presynaptic neurons. In turn, other enzymes that can hydrolyze 2-AG are ABHD6, usually located in dendrites and dendritic spines of excitatory neurons, and ABH12. Similarly, like AEA, 2-AG can also be metabolized by COX-2, and under certain conditions, FAAH could act in its degradation (19, 21, 23). Therefore, it could be hypothesized that COX-2 inhibitors may elevate basal levels of endocannabinoids, producing the beneficial effect observed.

On the other hand, the most popular phytocannabinoid is the delta-9-tetrahydrocannabinol (Δ9-THC), the main psychoactive component of Cannabis sativa cannabidiol (CBD), this one devoid of abuse and dependence, subject of intense medical research. CBD can bind to more than 65 targets and displays 100 times less affinity for CB1r than Δ9-THC (26, 27). In addition, one of the first synthetic treatments employed in humans that acts selectively to the EC system was the antagonist/inverse agonist of CB1r SR141716A (rimonabant). This compound was used to treat obesity, but its commercialization stopped due to a few cases of suicide reported in patients treated with this drug (19–21, 23, 24). This fact pointed out how the modulation of the endogenous cannabinoid system impacts psychiatric disorders.

## Cannabinoid Receptor 2 as a Potential Modulator of Neuroinflammation

**FIGURE 1:**
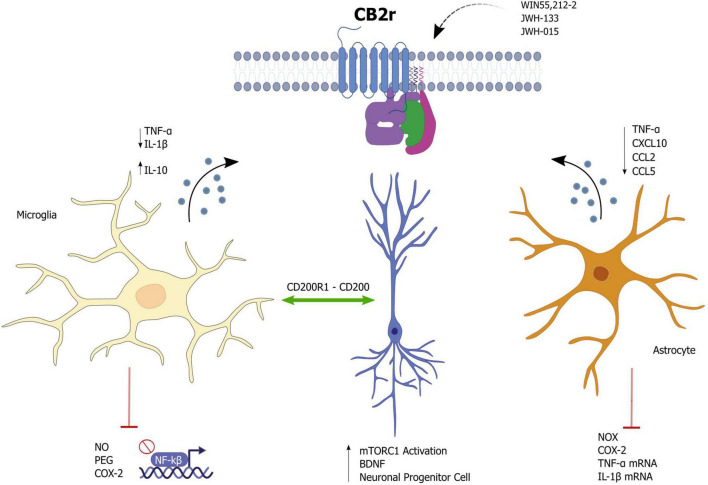
Effects of cannabinoid receptor 2 (CB2r) activation as a potential modulator of neuroinflammation.

Currently, CB2r are emerging as potential immunomodulatory agents with specific roles in cell-type specificity. Therefore, knowing these receptors will help understand the mechanisms by which CB2r could impact psychiatry due to the importance of inflammation in neuropsychiatric disorders. A large amount of studies have demonstrated the involvement of inflammatory mediators in mood disorders and in particular, IL-1β and TNF-α in behavioral alterations [For review see. Dantzer et al. (28), Remus and Dantzer (29), and Raison et al. (30)] For this reason, we will review in the following sections how the modulation of CB2 receptors shows promising results for the management of psychiatric diseases (Figure 1).

### The Involvement of the Cannabinoid Receptor 2 in the Microglia

Cannabinoid receptor 2 in microglia appears to be involved in a variety of paradigms and diseases involving neuroinflammation, including Alzheimer’s disease (AD), Parkinson’s disease, multiple sclerosis (MS), as well as stress or addiction (8, 31, 32). Some studies showed that activation of CB2 receptors prevents the activation of microglia and the release of pro-inflammatory mediators (33–35). This anti-inflammatory action seems to be responsible for a neuroprotective effect in different animal models of AD, MS, stroke, amyotrophic lateral sclerosis (ALS), and other diseases involving inflammation (36). However, the molecular mechanisms associated with these anti-inflammatory effects remain unknown.

Evidence suggests that CB2r controls microglial activity by interfering with the NF-κB pathway (37) and with the mitogen-activated protein kinases (MAPK) pathways [c-Jun N-terminal Kinase (JNK), ERK, or p38] (38). These kinases are well-known targets for the modulation of inflammation by interfering in downstream signaling pathways of the innate immune response and the production of inflammatory mediators (39). However, the activation of CB2r coupling to MAPKs is dependent on cellular context. A wide range of activation and inhibition has been observed depending on the cell type, cell differentiation status, and co-modulators of MAPK cascades (36).

Early reports showed that CB2r agonists prevented neuronal injury during neuroinflammation by regulating mitogen-activated protein kinase phosphatase-1 (MKP-1), resulting in Erk1/2 inhibition (34). Similarly, Romero-Sandoval reported that activation of CB2r with JWH-015, a selective CB2r agonist, induced an anti-inflammatory phenotype in microglia consistent with a reduction in TNF-α expression and a decrease in microglial migration through inhibition of ERK1/2 phosphorylation and MKP induction (40). Another study also found a reduction of p-ERK and p-p38 133 with an anti-inflammatory effect upon CB2r activation using JWH-133 (41). Moreover, oleamide (ODA), an amide fatty acid with actions at CB1 and CB2 receptors (42), showed a protective effect by inhibiting Nitric Oxide (NO), Prostaglandin E, COX-2 production through inhibition of Akt, p38, and ERK phosphorylation when BV2 microglia were treated with Lipopolysaccharide (LPS). This TLR4 agonist triggers inflammation through NF-kB (43). Guo et al. (37) also reported reduced levels of pro-inflammatory cytokines, such as IL-1β, TNF-α, and IL-6 in BV2 microglia following hypoxic exposure and treatment with Trans-caryophyllene. These anti-inflammatory effects were mediated by inhibiting the NF-κB pathway (37).

Noteworthy, Viscomi et al. (44) found that in axotomized neurons, the JWH-015 agonist did not produce neuroprotection via the ERK1/2 and p38 pathways during remote cell death. Instead, the beneficial effect was seen through an increase in p-Akt and a decrease in p-JNK, a PI3K-dependent effect, suggesting a protective effect of JWH-015 through regulation of JNK via the PI3K/Akt pathway. Importantly, there is a constitutive control of CB2r-mediated basal JNK activity in the mouse brain; the selective CB2r agonist JWH133 reduced JNK phosphorylation, whereas acute administration of the antagonist AM630 would increase it. In contrast, chronic administration of AM630 markedly decreases basal JNK activation, revealing a reversal of the acute effect of the antagonist (45).

In an *in vitro* model of multiple sclerosis, anandamide showed an anti-inflammatory action by inhibiting LPS/IFNγ-induced NF-kB activation. In particular, anandamide strongly activated the phosphorylation of ERK1/2 and JNK pathways while reducing Akt phosphorylation, which increased IL-10 production in microglia (46).

In addition, several reports suggest that the anti-inflammatory effect of CB2r activation might involve other different pathways such as JAK (Janus kinase)/STAT1 (signal transducer and activator of transcription) (35) or activation of the pCREB-Bcl-2 pathway (47).

CB2r has been positioned as an essential target mechanism for phenotypic change of microglia (38, 48). One of the main ideas of how CB2r can counteract inflammation is shifting microglia toward an anti-inflammatory phenotype, M2, with a consequent increase in the release of anti-inflammatory cytokines such as IL-10. Essentially, the endogenous endocannabinoids 2-AG and AEA increase the expression of the anti-inflammatory phenotype by microglia while decreasing M1 inflammatory microglia markers (46, 49). Similarly, palmitoylethanolamide (PEA) blunted the increase of M1 pro-inflammatory markers induced by lipopolysaccharide (LPS), concomitantly increasing those M2 anti-inflammatory markers in N9 microglial cells partially through CB2r activation (50).

The mechanisms involved in the switch of microglia to an anti-inflammatory phenotype by CB2r are the (cyclic AMP)-dependent protein kinase-mediated pathway found in *in vivo* and *in vitro* models of brain injury (51), the PI3K/Akt/mTOR/NF-kB pathway in neuroprotection induced in an animal model of vascular dementia (VD) (52), and the protein kinase C (PKC) and PGC-1α, a transcription protein involved in the regulation of mitochondrial biogenesis (50, 53).

Interestingly, the expression of cannabinoid receptors, mainly CB2, has been related to the microglial phenotype (49). For instance, results from *in vitro* studies suggest that the deletion of microglial CB2r leads to suppressing inflammatory phenotypes. Also, primary microglia from CB2r deficient mice are less responsive to pro-inflammatory stimuli (54). On the other hand, microglia from CB2r deficient mice cannot polarize to an M2a phenotype (49). Altogether, these findings suggest that CB2rs play a role in microglia activation either to a pro- or anti-inflammatory phenotype. Nevertheless, these findings are still ambiguous, and further research will clarify the fine-tune mechanisms involved in regulating pro- or anti-inflammatory behaviors associated with CB2.

Altogether, these data highlight the critical immunomodulatory functions of CB2 receptors in microglia. Moreover, modulation of this receptor may be an excellent strategy to target neuroinflammation in psychiatric diseases.

### The Involvement of the CB2 Receptor in Astrocytes

It is well-known the role of astrocytes at the level of neuronal support and metabolism (55). Still, it has also been seen how they can intervene, after systemic inflammation, in regulating cognitive functions (56). In this aspect, cb2 receptors expressed in the astrocytes could be contributing to the modulation of inflammation and metabolism in astrocytes to support neuronal function.

The treatment with the CB2r agonist WIN55,212-2, in IL-1β-stimulated human astrocytes, inhibited iNOS together with reduced release of pro-inflammatory mediators such as TNF-α, CXCL10, CCL2, and CCL5. Therefore, the authors suggested that it was primarily mediated by CB2r receptors (57). On the other hand, Jia et al. (58) showed that CB2r could mediate anti-inflammatory actions in astrocytes. CB2 receptor expression is upregulated in astrocytes treated with 1-methyl-4-phenylpyridinium (MPP+), a toxic metabolite that activates glial cells to release inflammatory mediators. Furthermore, the authors showed that pretreatment with the CB2r agonist JWH133 inhibits MPP+-induced iNOS and COX-2 protein up-regulation and TNF-α and IL-1β mRNA up-regulation in astrocytes. Moreover, the CB2r agonist inhibited MPP+-induced iron influx into astrocytes. The authors concluded that all these effects depended on CB2r (58). Additional anti-inflammatory effects for a CB2r agonist have also been reported in the context of spinal glial activation and pain sensitization (59). These findings suggest a functional relationship between astrocytic CB2r signaling and inhibition of p38 phosphorylation, which is associated with an anti-inflammatory effect and suppression of IL-1-induced CX3CL1 upregulation (60, 61).

In addition, pretreatment with a CB2r agonist protected against increased blood-brain barrier permeability in a model of spinal cord ischemia-reperfusion. This action may be primarily mediated by reduced expression of MyD88/NF-κB level pathway and the astrocytic TLR4/Matrix metallopeptidase 9 (MMP-9), an enzyme involved in BBB leakage (62). Other studies show how the stimulation with JWH133 alleviated neuroinflammation and protected BBB permeability in rat models of intracerebral hemorrhage (63, 64). In addition, CB2r is expressed in cell types, different from astrocytes, that form the blood-brain barrier, such as endothelial cells (65), showing the implication of the CB2r in the maintenance of the brain-periphery homeostasis.

### The Involvement of the CB2 Receptor in Neurons

The CB2 receptors are involved in protecting neuronal damage caused by neuroinflammation. The most studied mechanisms of this protection were those involving microglia. However, CB2 receptors are also expressed in neurons. This protective effect could be due to neuronal intracellular mechanisms, although it is unknown whether neuronal CB2r can modulate immune functions.

Marchant et al. showed how chronic activation of CB1 and CB2 receptors by WIN-2 proven an anti-inflammatory cytokine profile in the hippocampus of aged rats. While the agonist actions of WIN-2 at CB1/2 receptors promoted neurogenesis in the dentate gyrus, the antagonist actions of WIN-1 at the TRPV1 receptor were responsible for the reduction in microglial activation (66); Furthermore, CB2 receptors have been shown to promote neural progenitor cell proliferation through the activation of mTORC1 signaling (67).

To date, few studies have investigated the differential signaling pathways triggered by CB2r in microglial and neuronal cells. However, neuronal and microglial CB2 receptors have been shown to play different roles in behavioral regulation (68). One of the possible mechanisms that have been observed is the modulation of the transcription factor NRF2 through CB2 receptors. NRF2 is a master regulator of inflammation and modulates microglial dynamics. It has been shown to counteract inflammation in several neurodegenerative diseases by interacting with the transcription factor NF-κB. However, these findings have not been reproduced at the neuronal level (69). Therefore, this raises the possibility that CB2r in neurons has other functions not involved in the regulation of inflammation.

Another neuroprotective mechanism of CB2r is the bidirectional interaction between neurons and glial cells through the release of soluble factors and the formation of ligand-receptor associations. It is thought that CB2r-mediated modulation of neuron-glia crosstalk would be highly relevant for neuronal survival when brain homeostasis is lost. Recent studies have shown that MAGL inhibition increases the risk of neuronal death in neuronal cultures but not in neuron/microglia co-cultures. The authors conclude that the neuroprotective effect of MAGL inhibition was due to CB2r in microglia (70).

A possible protective mechanism involved in CB2r-mediated microglia-neuron interaction is the potentiation of the CD200-CD200R interaction. CD200R is expressed on microglia, whereas CD200 is expressed on neurons, and their interaction plays a crucial role in neuronal protection in inflammation-mediated neurodegeneration. Furthermore, CD200R1-CD200 interaction was associated with decreased production of the pro-inflammatory cytokines IL-1b and IL-6 but increased IL-10 in activated microglia after anandamide treatment (32). Another possible mechanism related to microglia-neuron interaction and CB2r involves the up-regulation of microglial CX3CR1. This effect promotes a neurotrophic phenotype of microglia, associated with increased release of BDNF by microglia and increased proliferation of neuronal progenitor cells (71).

Altogether, these data highlight an essential role for brain CB2r in modulating inflammatory responses. CB2r can modulate immune function by suppressing inflammation, shifting microglia to an M2 anti-inflammatory phenotype, supporting neuron-glia coupling, promoting neuronal proliferation, and neuroprotection, which makes them potential modulators for psychiatric conditions, as they present immune alterations. Importantly, data show an anti-inflammatory effect in multiple inflammatory diseases. Data regarding the molecular mechanisms are still uncertain since studies are performed in different conditions. However, these data reveal that CB2r can impact central routes involved in microglial functions (JNK, p38, ERK, Akt, mTOR). These pathways are also involved in neuropsychiatric disorders and inflammatory conditions. CB2r is emerging as a potential therapeutic target. Further studies are needed to reveal how CB2r mediates anti-inflammatory and neuroprotective actions in neuropsychiatric disorders.

## Contribution of Cannabinoid Receptor 2 in Modulating the Crosstalk Between Neuroinflammation, Neuroplasticity, and Emotional Disorders

### Cannabinoid Receptor 2 Modulation of Neuroinflammation in Depressive Disorders

Approximately 16% of the world population presents a depressive disorder, the third leading cause of disability and the fourth leading cause of disease burden (72). Its clinical features include a great variety of symptoms such as sleep disturbances, sadness, apathy, anhedonia, and reduced social interest. Depressive disorders are associated with a detriment in social relationships, including family breakdown, absence from work, and reduced productivity in the workplace. The prevalence of depressive disorders varies depending on age and sex, with the highest rates in women (73–76). Interestingly, the severity of depressive disorders is more significant at older ages (77).

Despite the significant number of antidepressants available, the clinical response rate is low, reflecting that only one-third of patients achieve complete remission (78, 79). The main reason for this clinical reality lies in the incomplete knowledge of the biological basis underlying depressive disorders, especially considering gender and age influence. Thus, it is necessary to invest more efforts and resources in characterizing in more detail the underlying mechanisms with the final goal of identifying new and effective critical targets for treating these disorders. In this respect, cumulative data pointed out CB2r as a promising therapeutic option for several neuropsychiatric conditions, including depressive disorders. The first evidence came from animal models of depression showing alterations of CB2r in different brain regions, such as the hippocampus (HIPP), amygdala (AMY), and frontal cortex (80–83). A postmortem study from the Manzanares group performed in the brain of suicide victims showed a reduction of CB2r gene expression in the dorsolateral prefrontal cortex and AMY, critical regions in decision making and impulsivity emotional reactivity (84). Besides, the research team led by Onaivi has shown a high incidence of the Q63R polymorphism of CB2r in Japanese depressive patients (85). The R allele of this functional polymorphism was also associated with increased sensitivity for childhood trauma, possibly due to a loss in the inhibition of inflammation and overactivation of the hypothalamic-pituitary-adrenal (HPA) axis (86).

In the same way, genetic and pharmacological studies performed in rodents further supported the role of CB_2_r in emotional reactivity, including anxiety and depressive-like behaviors. On the one hand, mice overexpressing CB_2_r in the central nervous system (neurons and glia) (CB2xP) presented decreased depressive-like behaviors in the tail suspension and novelty suppressed feeding tests (81). On the other hand, mice lacking CB_2_r (CB2KO) showed just the opposite since their vulnerability was higher in the evaluated paradigms (87). Overexpression of CB2r was associated with an endophenotype resistant to stimuli promoting a depressive state in the unpredictable chronic mild stress paradigm (UCMS), a reliable animal model to study the pathophysiological mechanisms underlying depression as decreased neurogenesis, HPA axis alterations and neuroinflammation (88–92).

Likewise, pharmacological studies chronically administering the cannabinoid CB_2_r-antagonist AM630 (1 mg/kg/12 h) revealed a reduction of depressive behaviors induced by CUMS (81). In contrast to these data, it was previously shown that administration of AM630 (3 mg/kg/24 h) produced no effect on sucrose consumption in CUMS-exposed mice (80). These discrepancies could be due to notable differences between the two studies, such as (1) the rodent strains employed [BALB/c (80), ICR (81)], and (2) the dose and administration patterns evaluated [3 mg/kg/24 h (80), 1 mg/kg/12 h (81)].

Based on the neuroinflammation hypothesis of depression (93–95), administration of the LPS, which induces sickness and depressive-like behaviors and cognitive impairments, is used to clarify the underlying mechanism of depression (96–98). A significant up-regulation of CB2r was identified in activated microglia using this model (99). Additional evidence about the role of CB2r in modulating neuroinflammation and depressive-like behaviors came from pharmacological studies testing CB2r-agonists. Acute and chronic administration (7 days) of the CB2r-agonist 1-phenylisatin (PI) significantly improved LPS-behavioral and neuroinflammation, reducing depressive-like behaviors, oxidative stress and TNF-α levels, and preventing the decline of antioxidant enzymes as GSH and catalase (100). Interestingly, Youssef et al. (101) observed that the CB2r-agonist beta-caryophyllene (BCP) alleviates insulin resistance, oxidative stress, neuroinflammation, and psychological changes, including depressive-like behaviors and memory deficits induced by high fat/fructose diet (HFFD) in male Wistar rats. More in detail, this study indicated that CB2r, together with PPAR-γ, is involved in the anti-inflammatory, anxiolytic and antioxidant effects of BCP. Further data supported anxiolytic- and antidepressant-like effects of BCP in different paradigms (102, 103). Interestingly, pre-administration of the CB2r-antagonist AM630 completely blocked BCP effects, supporting the involvement of CB2r in such anxiolytic and antidepressant properties (104). Curiously, blockade of CB2r by the antagonist SR144528 attenuated the effects of the synthetic cannabinoid HU210 (CB1r and CB2r agonist) on reducing the increase of cytokines concentrations induced by LPS in the brain (105).

Complementary studies revealed the involvement of CB2r in the antidepressant properties of different compounds. Such is the case of a very recent study showing that CB2r, along with CB1r and PPAR- γ, are involved in the antidepressant-like effects of the rosmarinic acid in the LPS-induced neuroinflammatory model (106). Besides, the compound terpineol, monoterpenoid alcohol, showed immunomodulatory, neuroprotective, and antidepressant-like effects mainly through CB1r and CB2r (107).

Interestingly, previous studies demonstrated the regulatory role of CB2r in stress-induced excitotoxicity and neuroinflammation in mice. JWH133 prevented the increase in pro-inflammatory cytokines and the cellular oxidative and nitrosative damage (lipid peroxidation) induced by immobilization and acoustic stress (2 h/day for 4 days). Similarly, CB2xP exhibited the same anti-inflammatory and neuroprotective actions as those observed in mice pretreated with JWH133. Conversely, CB2KO mice showed intensified stress-induced neuroinflammatory responses (108).

Additional studies also revealed a closed association between CB_2_r and classical monoaminergic systems involved in depression, such as the dopaminergic and the serotoninergic systems (109–111). More importantly, CB_2_r also influences neuroplasticity. In this respect, genetic mice models revealed alterations in BDNF, one of the main neurotrophic factors involved in neurogenesis, which plays an essential role in modulating the plasticity of adult neurons and glia cells (112). Overexpression of CB_2_r (Cb2xP) increased BDNF levels in the HIPP, which was not reduced even after 8 weeks of CUMS (81). In contrast, CB2KO presented low BDNF, MAP2, NF200 and SYN-immunoreactive fibers and a reduced number of synapses in the HIPP, supporting abnormalities in the dendritic reorganization and the synaptic connections (113). Indeed, chronic administration of AM630 significantly increases CB_2_r and BDNF gene expression in the HIPP of mice exposed to the CUMS, being proposed as a mechanism underlying its antidepressant properties in this mice model (81). These results strongly suggest that CB_2_r may play an essential role in regulating BDNF gene expression. Recent pieces of evidence supported how microglia can modulate neuronal plasticity potentially through BDNF. Thus, it is necessary to study further if modulation of microglia by CB2r may be the mechanism underlying the increase of BDNF observed in CB2xP mice.

Furthermore, the lack of CB2r (CB2KO) reduced the glucocorticoid receptor (GR) gene expression in the HIPP (113). Increased phosphorylation of proteins involved in the mTOR signaling pathway, p70S6K and AKT, was found in CB2KO, revealing potential alterations in the translational process that controls the protein synthesis underlying synaptic neuronal plasticity and memory (113–116). Considering the role of stress and GR in regulating inflammatory processes and BDNF gene expression (117, 118), it is tempting to speculate that the reduction of GR gene expression may cause changes in BDNF gene expression, neuronal plasticity and consequently alter the neurogenesis processes.

More recently, the neuroprotective effects of CB2r agonists were associated with the suppression of microglial activation through the inhibition of neurotoxic factors and by decreasing neuronal cell damage (119). During CNS inflammation, activation of CB2r inhibited ERK-1/2 signaling in microglia, reducing iNOS production (34). Studies carried out with the BCP demonstrated that this CB2r-agonist presents a modulatory effect on the PGC-1α/BDNF pathway (103). Additional pharmacological studies using CB2r-agonists, such as JWH133 and AM1241, showed that activation of CB2r significantly upregulated BDNF while reducing a variety of neuroinflammatory markers (50, 120). Altogether revealed that CB2r, through the regulation of microglial activity, modulates BDNF gene expression and, consequently, neuroplasticity.

It is important to note that cumulative evidence highlight that the success of antidepressant treatments lies in their ability to reduce inflammatory processes and improve trophic factors (121, 122). Moreover, recent studies have demonstrated that antidepressants promote microglial phenotype switching to M2-like cells, which can secrete anti-inflammatory cytokines, an effect that is also found by activation of CB2r on these cells (123, 124). Interestingly, microglia show sex- and age-differential characteristics. Aging female microglia selectively upregulates M1 markers with a poor response to noxious and stress stimuli (125). These results indicate the CB2r as a promising target for treating depressive disorders that deserve further exploration, especially considering sex and age. For summarized data see Table 1.

**TABLE 1:** Summary of the modulatory effects of cannabinoid receptor 2 (CB2r) on neuroinflammation in depressive disorders.

Genetic or pharmacological approach	Type	Strain	Paradigm	Behavioral effects	Neurochemical alterations	References
CB2r-agonist	PI (20 mg/kg; i.p. acute/chronic administration	Swiss Albino mice	LPS (1.5 mg/kg; i.p.)	Improve locomotor ↓ depressive-like behaviors	In brain homogenates: ↓ oxidative stress (MDA) ↓ TNFα	(100)
	BCP (acute; 30 mg/kg; p.o.)	Wistar rats	HFFD	↓ Anxiety and ↓ depressive-like behaviors. ↓ memory.	In the PFC: ↓ NOS-2, TNF-α and NF-κB levels	(101)
	BCP (chronic; 25, 50 or 100 mg/kg/24 h; p.o.)	Sprague-Dawley rats	Chronic stress	↓ Depressive-like behaviors	In the HIPP: ↑ BDNF (all doses) ↓ COX-2 (50 and 10 mg/kg) ↑ CB2r (25 and 50 mg/kg)	(103)
	HU210 (acute; 100 μg/kg; i.p.)		LPS (100 μg/kg; i.p.)	–	↓ IL-1β in the PFC, cortex, hypothalamus, amygdaloid cortex, thalamus, and cerebellum at 2 h of LPS administration ↓ IL-1β in the PFC, amygdaloid cortex, thalamus and cerebellum at 4 h of LPS administration ↓ TNFα in the PFC, thalamus and cerebellum at 2 h of LPS administration ↓ TNFα, IL-1β, IL-6 and INFγ in plasma	(105)
	JWH133 (2 mg/kg, i.p., 4 days)	Swiss Albino mice	Immobilization and acoustic stress (2 h/day for 4 days)	–	In FC: ↓ pro-inflammatory cytokines (TNFα and CCL2) ↓ cellular oxidative (NF-kB, NOS-2, COX-2)	(108)
KO mice	CNR2					
Transgenic mice						

*↓, decrease; ↑, increase; –, not measured/evaluated or not applied.*

### Cannabinoid Receptor 2 Modulation of Neuroinflammation in Anxiety Disorders

Anxiety disorders are the most common mental diseases, affecting nearly 30% of adults at some point in their lives (126). They present high comorbidity with other psychiatric disorders, among which depressive disorders stand out (73, 127, 128). Different factors have increased the likelihood of developing anxiety, such as genetic, environmental, psychological, and developmental causes (129–135). The treatment of anxiety disorders includes psychotherapy and pharmacotherapy such as anxiolytic and antidepressant drugs. Among anxiolytics, benzodiazepines (BZP) are the most common, generally prescribed only for short periods. Selective serotonin reuptake inhibitor (SSRI) and serotonin and norepinephrine reuptake inhibitor (SNRI) antidepressant classes are the first-line medication treatments (136, 137). Despite all the therapeutic options available, their limited efficacy and side effects (138–143) prompted further research to elucidate the underlying mechanisms. Therefore, identifying new targets and drugs alone with current pharmacological options would improve clinical effectiveness and safety outcomes.

The identification of CB2r in brain regions is closely related to the response to stress, as the HIPP and AMY (81, 144, 145) accelerated the development of studies to clarify its role in modulating anxiety. In rodents, exposure to different types of stressful stimuli induced significant changes in the expression of CB2r in the brain. Such is the case of maternal deprivation (MD), a model used for evaluating long-lasting effects of early life stress, including anxiety- and depressive-like behaviors, cognitive impairments and neuroendocrine alterations (146–148). In rats, MD significantly increased the expression of CB2r in the HIPP (149). Similarly, additional studies revealed sex-dependent differences, observing an increase of CB2r gene expression in the frontal cortex, ventral and dorsal striatum, dorsal HIPP and AMY in MD-male rats. In females, such an increase was only observed in the HIPP, suggesting that consequences of early life stress depend on sex and brain region (83). Moreover, a rapid increase in CB2r gene expression has been observed in the HIPP of mice exposed to social defeat (150).

Studies using mice modified genetically demonstrated that CB2xP showed resistance to anxiogenic-like stimuli in the light-dark box and elevated plus maze tests (151). On the contrary, CB2KO mice presented anxious behaviors (87). More interestingly, genetic manipulation studies allowed us to go more deeply into the cell-specific active involvement of CB2r in the HIPP, dissecting the effects of CB2r gene expression disruption or overexpression in hippocampal neurons or microglia in the regulation of anxiety behavior and cognition (68). The results revealed that overexpression of CB2r in CA1 pyramidal neurons significantly reduced anxiety levels. In the case of microglia, the elevation of CB2r increased contextual fear memory, whereas the absence of CB2r induced a reduction. Moreover, the deletion of CB2r in VTA dopaminergic neurons caused a significant anxiolytic-like effect (152). Altogether, these results revealed that CB2r plays distinct roles in regulating anxiety and memory depending on the type of cells expressed.

Pharmacological studies also revealed the role of this cannabinoid receptor in stress response and anxiety. Acute treatment with the CB2r-agonist JWH015 reduced anxiety-like behaviors in stressed mice; however, its chronic administration induced opposite effects (153). In line with these results, acute activation of CB2r by BCP caused an anxiolytic-like effect entirely abolished by the CB2r-antagonist AM630, supporting the role of CB2r on BCP effects (102, 104). Alternatively, it has been demonstrated that chronic administration of the CB2r-antagonist AM630 reduced the anxious behaviors induced by the CUMS (81). Similarly, chronic intracerebroventricular administration of the CB2r antisense oligonucleotide induced anxiolytic-like effects (154). Although additional studies are needed, the data collected to the date indicated potential differences between acute and chronic administration effects of compounds acting on CB2r. The CB2r agonists appear to be more helpful in producing an acute anxiolytic effect, whereas CB2r antagonists would be more appropriate for chronic anxiolytic treatments. CB2r has been closely related to crucial targets in response to stress and anxiety, including the HPA axis (151), the GABAergic system (151, 155), and more recently, neuroinflammation. The modulation of neuroinflammatory elements was associated with the anxiolytic-like effects of the non-selective CB2r-agonist WIN55,212-2 in mice exposed to repeat social deficits. This cannabinoid compound decreased IL-1β gene expression in microglia/macrophages and the accumulation of peripheral inflammatory monocytes (156). Interestingly, in animal models of traumatic brain injury, the modulation of neuroinflammation by CB2r agonist reduced the development of anxiety and depressive-like behaviors in rodents (157–159). Thus, modulation neuroinflammation appears to be a mechanism by which CB2r modulates emotional responses deserving an in-depth exploration (Table 2).

**TABLE 2:** Summary of the modulatory effects of cannabinoid receptor 2 (CB2r) on neuroinflammation in anxiety disorders.

Genetic or pharmacological approach	Type	Strain	Paradigm	Behavioral effects	Neurochemical alterations	References
CB2r agonist	WIN55,212-2 (1 mg/kg; i.p.; 6 days; prior to each cycle of RSD)	C57BL/6 mice	Repeat social defeat	↑ Anxiety-like behaviors in the EPM and fear conditioning	↓ IL-1B in microglia/macrophages and the accumulation of peripheral inflammatory monocytes	(156)
	O-1966 (5 mg/kg; i.p.; subchronic)		Traumatic brain injury	↓ Motor alterations in the OF and rota-rod	↓ Blood-brain barrier permeability ↓ neuronal degeneration (Fluoro Jade C labeling in the somatosensory cortex) ↓ macrophage/microglia cell counts (Iba-1 + cells) in the injury hemisphere	(159)

*↓, decrease; ↑, increase; –, not measured/evaluated or not applied.*

## Contribution of Cannabinoid Receptor 2 in Modulating the Crosstalk Between Neuroinflammation, Cognitive Dysfunction, and Neurodegeneration

The role of CB2r in the onset of Alzheimer’s disease (AD) has been intensively investigated in the last decades. Inflammation has been shown to prompt cognitive dysfunction and dementia later in life (160). Several studies associate depression with a high risk of developing dementia, and often those diseases are concomitant (161).

Unraveling its pathophysiological role in AD can provide a new therapeutical strategy. CB2r are poorly expressed in the neuronal brain cells in healthy conditions and are primarily detected in glial cells. However, CB2r levels drastically increase in neurodegenerative disorders, playing an essential role in modulating pro-inflammatory mediators such as AD (162, 163). High levels of CB2r have been found in senile plaques surrounding microglia and astrocytes in both cortex and hippocampus in postmortem human AD patients (164, 165). Similarly, in a preclinical murine AD model, a high expression of CB2r was also found in astrocytes and microglia surrounding the amyloid plaques but not neuronal cells (166). Interestingly, expression levels of CB2r correlated with two relevant AD molecular markers, Aβ42 levels and senile plaque score. Both human and rodents’ findings support a potential role of CB2r in the inflammatory response generated by plaque deposition.

Although most studies found a correlation of CB2r expression and Aβ42 levels, Tau overexpression appears to be involved in modulating CB2r levels in both mouse and human brains (167). This study showed a TAU-dependent increase of CB2r expression on a neuronal level, and most importantly, the deletion of CB2r in TAU mice was associated with a cognitive improvement. However, it was ineffective to reverse neuroinflammation supporting a neuronal CB2 effect in TAU mice rather than glial, as reported in APP Tg AD mouse models. To further investigate the role of CB2r, Aso et al. (168) produced an AD(APP/PS1)/CB2 KO transgenic mice and evaluated the specific contribution of CB2r in the AD pathology. The deletion of the CB2r increased cortical Aβ deposition and Aβ40 soluble levels on the APP/PS1 mice. However, no effect was reported on tau pathology. Similar results were also previously observed in the mice model J20APP/CB2 KO (169). In addition, in this study, the deletion of CB2r reduced total Tau expression, supporting a divergent effect of CB2r between the Aβ and Tau pathology. In other studies, in contradiction with Aso’s et al. (168) findings, CB2r deletion in APP/PS1 mice was associated with improved cognitive and learning deficits. These findings were accompanied by reduced neuronal loss, decreased plaque levels, and a reduction of activated microglia (170).

CB2r has been a target of several *in vitro* and *in vivo* tests in preclinical studies, sometimes contradictory results. In primary rat hippocampal neuronal cultures, it has been shown that the CB2r agonist JWH133 treatment reduces the Aβ42–induced neuron apoptosis (171), reverted effect by the selective CB2r antagonist AM630. CB2r activation with the selective CB2r agonist JWH-015 suppressed IFN-γ-induced microglia activation, TNF-α, and nitric oxide production (35). In APP/PS1 mice, the same compound enhanced the novel object recognition memory deficiency but was inefficient for hippocampus-dependent spatial cognitive dysfunction in the Morris water maze test. Moreover, activation of CB2r did not affect plaque deposition. Pretreatment with MDA7, a selective CB2r agonist, in an Aβ42 induced AD-Murine model, was associated with a reduction in microglia activation and pro-inflammatory IL-1β production. An improvement in memory impairment was found in the Morris water maze test (172). Recently, similar results were reported after treating APP/PS1 mice with anandamide analog (NITyr). NITyr treatment improved motor coordination spatial memory and reduced Aβ40 and Aβ42 without affecting the APP expression (173).

Administration of CB2r agonists may affect microglia-dependent neuroinflammation. In addition, CB2r activation over CB1r compounds results in a more appealing therapeutic strategy since CB2r activation is not linked to the psychoactive effects linked to CB1r activation. Taken together (Table 3), these findings, and considering the high levels of CB2r in microglia observed in preclinical and clinical studies, CB2r can be regarded as a novel target for AD therapy.

**TABLE 3:** Summary of the modulatory effects of cannabinoid receptor 2 (CB2r) on neuroinflammation in cognitive dysfunction and neurodegeneration.

Genetic or pharmacological approach	Type	Strain	Paradigm	Behavioral effects	Neurochemical alterations	References
CB2r agonist	JWH-015	Microglial Cell culture, from BALB/c mice	–	–	Suppressed IFN-γ induced microglial activation. ↓ TNF-α, NO	(35)
KO mice	CNR2	C57BL/six mice	–	↑ Cognition.	It was ineffective to reverse neuroinflammation	(168)
			–	–	Microgliosis not differ between groups	(169)
			APP/PS1 transgenic mice	↑ Cognitive and learning deficits	Microglia with more ramifications and smaller and condensed plaques than APP/PS1 mice	(170)
CB2r agonist	MDA7 Subchronic (15 mg/kg; i.p)	Sprague–Dawley rat	Amyloid-induced memory deficiency	↑ Novel object recognition memory	↓ IL-1β	(172)
	NITy (15, 30, and 60 mg/kg; p.o) chronic	C57BL/J mice	APP/PS1 transgenic mice	↑ Recovery learning and memory abilities in Morris Water Maze	Neuroprotective effect inducing autophagy	(173)

*↓, decrease; ↑, increase; –, not measured/evaluated or not applied.*

## Conclusion

Studies developed to the date indicated that CB2r is involved in emotional response and cognition. Its pharmacological modulation could be an exciting tool to treat different neuropsychiatric disorders. Despite the correlation between the immune system and the brain, the implication of CB2r needs to be further elucidated. CB2r expression in cells of immune systems points out the possible involvement of this system in psychiatry and neurology with the implication of CB2r. Here, different actions have been related to CB2r and its pharmacological modulation, including changes in inflammatory pathways and microglial functions associated with NF-κB, JAK, MAPK, and AKT signaling pathways. Remarkably, a neuroprotective action has been proposed for this receptor, possibly by producing a shift of microglia toward an anti-inflammatory phenotype. In addition, CB2r modulation also changes the response of astrocytes and neurons on inflammation, protecting from BBB leakage or promoting neural plasticity. Consequently, the CB2r modulation may be a promising target to improve neuropsychiatric diseases associated with neuroinflammation.

Several human clinical trials are ongoing to look at the potential modulation of the cannabinoid system in human disease. However, no studies are currently underway with selective modulation of the CB2r to treat neuropsychiatric illnesses. Among the following strategies, we find the use of FAAH inhibitors, for instance, using the compound PF-04457845 in Tourette syndrome (NCT02134080) or cannabis use disorder (NCT03386487). The potential use of URB597 for the treatment of schizophrenia has been shown to protect against NLRP3 inflammasome activation (97). However, no studies with MAGL inhibitors are currently underway. Furthermore, the potential use of CBD, directly modulating the EC system, to treat anxiety (NCT02548559), psychosis (NCT03883360), as well as the anxiety and agitation symptoms in mild to moderate Alzheimer Disease (NCT04075435), are currently being evaluated. Besides, the use of nabilone, an analog of THC, is also being studied for non-motor symptoms in patients with Parkinson’s Disease (PD) (NCT03773796).

It is unclear whether the therapeutic effect could be due to the improvement of inflammation through modulation of the CB2r or other critical targets of the ECS. Therefore, the specific role of the CB2r in inflammation associated with psychiatric disorders requires further study.

## Author Contributions

AM and TF designed, coordinated, and reviewed the sections and contents of the review manuscript. TF oversaw the organization to distribute the writing tasks among the authors and the article writing. AM, MSG-G, ST, and EN performed the literature searches. AM, MSG-G, ST, JM, and TF participated in the manuscript writing. All authors critically reviewed and approved the final version of the manuscript.

## Conflict of Interest

The authors declare that the research was conducted in the absence of any commercial or financial relationships that could be construed as a potential conflict of interest.

## Publisher’s Note

All claims expressed in this article are solely those of the authors and do not necessarily represent those of their affiliated organizations, or those of the publisher, the editors and the reviewers. Any product that may be evaluated in this article, or claim that may be made by its manufacturer, is not guaranteed or endorsed by the publisher.
